# Identification of genomic copy number variations associated with specific clinical features of head and neck cancer

**DOI:** 10.1186/s13039-018-0354-8

**Published:** 2018-01-15

**Authors:** Boris Zagradišnik, Danijela Krgović, Špela Stangler Herodež, Andreja Zagorac, Bogdan Ćižmarević, Nadja Kokalj Vokač

**Affiliations:** 1 Laboratory of Medical Genetics, University Medical Centre Maribor, Ljubljanska 5, 2000 Maribor, Slovenia; 2 Department of Otorhinolaryngology, University Medical Centre Maribor, Ljubljanska 5, 2000 Maribor, Slovenia

**Keywords:** Head and neck cancer, Copy number variations, Array comparative genomic hybridization, Biomarkers

## Abstract

**Background:**

Copy number variations (CNSs) of large genomic regions are an important mechanism implicated in the development of head and neck cancer, however, for most changes their exact role is not well understood. The aim of this study was to find possible associations between gains/losses of genomic regions and clinically distinct subgroups of head and neck cancer patients.

**Results:**

Array comparative genomic hybridization (aCGH) analysis was performed on DNA samples in 64 patients with cancer in oral cavity, oropharynx or hypopharynx. Overlapping genomic regions created from gains and losses were used for statistical analysis. Following regions were overrepresented: in tumors with stage I or II a gain of 2.98 Mb on 6p21.2-p11 and a gain of 7.4 Mb on 8q11.1-q11.23; in tumors with grade I histology a gain of 1.1 Mb on 8q24.13, a loss of a large part of p arm of chromosome 3, a loss of a 1.24 Mb on 6q14.3, and a loss of terminal 32 Mb region of 8p23.3; in cases with affected lymph nodes a gain of 0.75 Mb on 3q24, and a gain of 0.9 Mb on 3q26.32-q26.33; in cases with unaffected lymph nodes a gain of 1.1 Mb on 8q23.3, in patients not treated with surgery a gain of 12.2 Mb on 7q21.3-q22.3 and a gain of 0.33 Mb on 20q11.22.

**Conclusions:**

Our study identified several genomic regions of interest which appear to be associated with various clinically distinct subgroups of head and neck cancer. They represent a potentially important source of biomarkers useful for the clinical management of head and neck cancer. In particular, the *PIK3CA* and *AGTR1* genes could be singled out to predict the lymph node involvement.

## Background

The head and neck squamous cell carcinomas (HNSCCs) are among the most common cancers, affecting approximately 530,000 new patients and causing 293,000 deaths worldwide every year [1]. The overall survival rate of around 50% is an important indicator that this type of cancer represents a major medical problem [2]. An improvement in disease management can be expected from better understanding of molecular mechanisms underlying this medical condition. Acquired DNA mutations which range from epigenetic DNA methylation alterations, single nucleotide changes to variations in whole chromosomal copy number are a well-established cause of cancer. An important subset represent copy number variations (CNVs) which can include large chromosomal regions or gene-size changes [3–5]. Gains and losses of particular genomic segments usually contain important oncogenes and tumor suppressor genes. These include such regions as a gain of 11q13 (*CCND1* gene, cyclin D1), a gain of 7p11 (*EGFR* gene, epidermal growth factor receptor), a loss of 9p (*CDKN2A* gene, cyclin dependent kinase 2a / p16), among many others [3, 6, 7]. The development of array comparative genomic hybridization provided further evidence for the role of frequent and characteristic gains and losses in HNSCCs. The accumulating data clearly reveal great heterogeneity apparent from a long list of genomic regions harboring gains (1q, 3p, 3q, 5p, 7p, 7q, 8q, 9q, 11q, 14q, 16p, 19q, 20q) and losses (2p, 3p, 3q, 4q, 8p, 10p, 16q, 18q) [8–11]. These are all excellent tumor markers which when present clearly and unequivocally identify a tissue sample as cancerous. However, the adoption of CNV detection for other roles in disease management is not easy because data supporting associations with various clinical parameters are often sparse or even contradictory [12, 13]. Consequently, these genetic biomarkers remain the interest of research with few of them nearing clinical practice of head and neck cancer treatment [14]. Nevertheless, they are of great importance in a quest to refine the current clinical description of tumors based on the TNM classification [15]. High heterogeneity exhibited by HNSCC may not always be sufficiently accounted for by the disease staging and pathomorphologic characterization. Therefore, it is important to continue the search for additional parameters which may help improve the categorization of individual cases of HNSCC. A hypothesis-free whole genome analysis is the modern approach to conduct such an examination [16].

This study identifies possible associations between gains/losses of genomic regions and clinical features characteristic for two distinct subgroups of head and neck cancer patients, those with early disease vs. those with loco-regionally advanced cancer.

## Methods

### Patients

The study included 64 patients (50 males, 78.1%; 14 females, 21.9%) with the diagnosis of head and neck cancer located in oral cavity, oropharynx or hypopharynx, who underwent treatment at the Department of Otorhinolaryngology, Cervical and Maxillofacial Surgery, University Medical Centre Maribor, Slovenia. They were recruited between November 2010 and March 2015. Clinical information about age, gender, age at diagnosis, follow-up and survival time, and disease recurrence was collected from medical records. The stage of the disease was determined from the TNM status of the tumor; however, not all patients received surgical treatment and postoperative evaluation was not available for all tumors. Histologic grade was also obtained. In addition, the treatment regimens in patients were recorded as surgery, chemotherapy, radiotherapy or the combinations thereof.

This study was approved by the Ethics committee of the University Medical Centre Maribor, where the study was conducted. Each patient signed an informed consent prior to the study enrollment.

### Tumor samples

Tumor samples were excised from primary tumor sites and not from metastatic sites or affected lymph nodes. All samples were specimens for pathological confirmation following surgery or diagnostic biopsy of which a small part was obtained for the long term storage at −80 °C.

### DNA extraction

At least 10 mg of tumor tissue was thoroughly disrupted with the TissueLyzer (Qiagen G.m.b.h., Hilden, Germany). The lysate was then used for the extraction of genomic DNA with the Qiaamp DNA Mini Kit on QIACUBE (both Qiagen G.m.b.h., Hilden, Germany). All procedures were performed according to the manufacturer’s instructions.

### Human papilloma virus detection

In order to ascertain the presence of human papilloma virus (HPV) DNA in tumor samples a simple duplex polymerase chain reaction protocol was developed. A 10 μL reaction contained 10 μM of primers MY09 (5’-CGTCCMARRGGAWACTGATC-3′) and MY11 (5’-GCMCAGGGWCATAAYAATGG-3′) [17], 1 μM of control locus primers (Forward: 5’-CTATCCCACTGTATTATTCAGGGC-3′; Reverse: 5′-TGAGTCTCCAGGTTGCAGGTGACA-3′), 50 ng of tumor genomic DNA in 1× Multiplex PCR Master Mix Kit (Qiagen G.m.b.h., Hilden, Germany). The temperature protocol was: 15 min of initial denaturation/enzyme activation at 95 °C, followed by 35 cycles of 30 s denaturation at 94 °C, 1 min annealing at 55 °C and 1 min elongation at 72 °C. The amplification products were detected with agarose gel electrophoresis after staining with SYBR Green I.

### Array comparative genomic hybridization analysis

Array CCH analysis was performed using the BlueGnome Cytochip lSCA 8x60K platform (BlueGnome Ltd., Cambridge, United Kingdom). The assay was performed according to the manufacturer’s instructions. Data were obtained using the BIueFuse Multi software tool. Automatic assessment provided by the software was used to acquire genetic variations. A minimal size of 20,000 base pairs (20kbp) was adopted as an inclusion criterion. The data were arranged according to chromosomal location to obtain the extent and frequency of overlapping regions, which were then used for further analysis. Gains and losses were analyzed separately. The GRCh37/hg19 was used for genomic data analysis and representation.

### Statistical analysis

The odds ratios (ORs) and chi-squared test (Chi2) with Yates correction were used to evaluate differences in copy number variation frequencies stratified by the patients’ characteristics. Patients were grouped according to the disease stage (stage I & II vs. stage III & IV), histological grade (grade 1 vs. grade 2 & 3), tumor size (T1 & T2 vs, T3 & T4), lymph node involvement (cases N0 vs. cases N1 or higher) and surgical treatment (surgery used vs. no surgery used). A 2 × 2 contingency table was generated between each of the five clinical feature groups and each genomic region involved. 95% confidence intervals (CI) were computed where applicable and a *p* value <0.05 was considered statistically significant. All analyses were done in OpenOffice, org Calc spreadsheet application.

## Results

Our study included 64 patients, mostly men (50, 78.1%, male to female ratio 3.57:1). with similar median age at diagnosis for both gender groups (Table 1). Since the study enrollment 42 patients have died (33 due to cancer, 9 from other causes) and 3 were lost to follow-up. Histologically verified squamous cell carcinoma was present in all patients. Most patients were affected with HNSCC for the first time; however, in 3 patients the cancer has re-occurred after a prolonged disease free interval. The tumors were positioned in oral cavity (34 patients), oropharynx (25 patients), and hypopharynx (5 patients) (Table 1), although for some large tumors their locations could not be precisely determined because they affected multiple adjacent anatomic regions. Most cancers were locally advanced (47 stage IV cases) and lymph node involvement was also frequent (42 cases) (Table 1). Distant metastases were a rare event observed in 3 cases (two in the lung; one in the brain) (Table 1). Various combinations of radical surgery, radiotherapy and chemotherapy were used for cure or palliative care in 62 patients, whereas in 2 patients their very poor general condition precluded the treatment of cancer (Table 1).

**Table 1:** Clinical data of patients with HNSCC

	# (%)	Median age at diagnosis (range)		Median observation time (range)	
Women	14 (21.9%)	56.5 (48–76) years		13 (2–53) months	
Men	50 (78.1%)	57 (34–78) years		22 (2–61) months	
Deceased	42 (65.6%)				
Status unknown	3 (4.7%)				
Disease reoccurrence	3 (4.7%)				
Tumor locations	# (%)				
Oral cavity	34 (53.1%)				
Oropharynx	25 (39.1%)				
Hypopharynx	5 (7.8%)				
TNM classification					
Tumor	# (%)	Lymph node status	# (%)	Distant metastases	# (%)
T1	8 (12.5%)	N0	22 (34.4%)	M0	61 (95.3%)
T2	16 (25.0%)	N1 or higher	42 (65.6%)	M1	3 (4.7%)
T3	7 (10.9%)				
T4	33 (51.6%)				
Disease stage	# (%)		Histologic grading	# (%)	
I	6 (9.4%)		grade 1	12 (18.7%)	
II	8 (12.5%)		grade 2	36 (56.3%)	
III	3 (4.7%)		grade 3	16 (25.0%)	
IVA	37 (57.8%)				
IVB	7 (10.9%)				
IVC	3 (4.7%)				
Cancer treatment applied	# (%)				
Radical surgery	13 (20.3%)				
Radical surgery & Chemotherapy & Radiotherapy	13 (20.3%)				
Radical surgery & Radiotherapy	18 (28.1%)				
Chemotherapy & Radiotherapy	10 (15.6%)				
Radiotherapy	8 (12.5%)				
No therapy	2 (3.1%)				

The aCGH analysis detected a total of 689 CNVs, 438 (63.6%) were identified as gains (additional copies of genetic material present) and 251 (33.4%) were losses (deficit of genetic material). Most variations were classified by the BlueFuse Multi software as potentially pathogenic, 483 out of 689 (70.1%), whereas 50 variations (7.3%) were declared as benign CNVs and the rest, 156 (22.6%) as unknown. The size of variations ranged between the cut-off value of 20 kb and whole chromosomes. The level of gain/loss, the log2 ratio, also included a wider range of values, which is clearly the consequence of the presence of tumor DNA with substantial amount of somatic mosaicism. Only 2 samples have not yielded any variations. Summarized data are presented in Table 2.

**Table 2:** Summary data on observed copy number variations

	#	Pathogenic (%)	Benign (%)	Unknown (%)	Log2 range	Size range (bp)
Gains	438	321 (73.3%)	24 (5.5%)	93 (21.2%)	0.22 -- 2.82	20,486–155,171,772
Losses	251	162 (64.5%)	26 (10.4%)	63 (25.1%)	−3.62 -- -0.25	23,615–127,293,063

2/64 samples without CNVs: male, oropharynx, stage IVA, pT4pN2, survival time 45 months; female, tongue, stage II, pT2pN0, alive after 40 months

The association analysis between overlapping genomic regions constructed from obtained variations and stratified clinical data revealed several statistically significant results (Table 3). Each genomic region statistical data is complemented with the range of Log2 ratios values as well as sizes of CNVs that overlap at the particular region. In low stage tumors (stage I and II) a 2.98 Mb region from 6p21.2-p11 and a 7.4 Mb region from 8q11.1-q11.23 were overrepresented in comparison to higher stage tumors. Within the latter locus a smaller 1.4 Mb genomic region has a higher association (Table 3).

**Table 3:** Genomic regions associated with specific clinical features in HNSCC patients

Stage I&II vs III&IV							
	Locus	#	OR (95% CI)	p(Chi2)^a^	Log2 ratio range	CNV sizes^c^	# of genes
gain 1	chr6: 38,729,522–41,698,277	3/11 vs. 0/50	–	0.084	0.54–0.62	18.6–19.5 Mb	35
gain 2	chr8: 46,942,986–54,391,986	4/10 vs, 2/48	0.104 (0.017–0.649)	0.0232	0.29–0.9	1.56–146 Mb	20
gain 3	chr8: 49,091,330–50,447,801	6/8 vs. 3/47	0.085 (0.018–0.411)	0.0021	0.29–0.9	1.56–146 Mb	5
Histological grade 1 vs. grade 2 & 3							
	Locus	#	OR (95% CI)	p(Chi2)^a^	Log2 ratio range	CNV sizes^c^	# of genes
gain 4	chr8: 122,449,344–123,538,355	5/7 vs. 6/46	5.476 (1.312–22.851)	0.0385	0.28–0.58	1.0–145 Mb	2
loss 1^b^	chr3: 93,979–80,817,252	5/7 vs. 2/50; 4/8 vs. 3/49	17.86 (2.891–110.3) 8.167 (1.532–43.52)	0.0011–0.024	−0.64 – −0.26	1.4–93 Mb	647
loss 2	chr6: 85,683,523–86,925,006	2/10 vs. 0/52	–	0.0384	−0.7 – −0.26	1.24–40 Mb	6
loss 3	chr8: 221,641–32,847,282	3/9 vs. 1/51	17 (1.587–182.1)	0.0205	−0.64 – −0.26	6.8–43 Mb	309
Node lymph involvement vs. cases without affected lymph nodes							
	Locus	#	OR (95% CI)	p(Chi2)^a^	Log2 ratio range	CNV sizes^c^	# of genes
gain 5	chr3: 147,963,947–148,708,961	9/33 vs. 0/22	–	0.0495	0.28–0.96	5.6–109 Mb	3
gain 6	chr3: 178,764,078–179,689,394	14/28 vs. 1/21	9.414 (1.141–77.65)	0.0349	0.27–0.96	5–109 Mb	13
gain 7	chr8: 108,460,956–109,567,888	3/39 vs. 7/15	0.165 (0.038–0.723)	0.0264	0.28–0.8	0.2–146 Mb	4
Cases treated with surgery vs. cases without surgical treatment							
	Locus	#	OR (95% CI)	p(Chi2)^a^	Log2 ratio range	CNV sizes^c^	# of genes
gain 8	chr7: 92,828,329–105,027,875	2/42 vs. 5/15	0.143 (0.025–0.816)	0.0457	0.35–0.83	21–78 Mb	196
gain 9	chr20: 33,690,620–34,021,768	0/44 vs. 3/17	–	0.0462	0.35–0.67	33-34 Mb	9

^a^degree of freedom = 1

^b^values in categories # and OR correspond to the p(Chi2) values which are the lower and upper boundary of observed statistical significance in this region; values in categories Locus, Log2 ratio range and CNV size are range boundaries that do not correspond to the displayed statistical data for the entry loss1

^c^column CNV sizes displayes the range of sizes of CNVs that include the region of each particular entry

A 1.1 Mb region on 8q24.13 was overrepresented in well-differentiated tumors (grade 1) compared to poorly differentiated tumors (grades 2 & 3). The loss of a large part of a short arm of chromosome 3 was significantly more common in grade 1 tumors. This approximately 80 Mb long continuous region from 3p was found to be associated with a varying degree of statistical significance as indicated in Table 3 (entry loss 1). The data for this genomic region are displayed only as ranges includeing maximal and minimal values from all smaller overlapping genomic regions that constitute the entire entry loss 1. The loss of further genomic regions associated with well differentiated tumors included a 1.24 Mb region on 6q14.3 and a 32 Mb terminal region of chromosome 8 (8p23.3).

Furthermore, genomic regions that were significantly overrepresented in cases with lymph node involvement were a gain of 0.75 Mb region on 3q24 and a gain of 0.9 Mb region on 3q26.32-q26.33. Genomic regions overrepresented in cases without lymph node involvement included a gain 1.1 Mb region on 8q23.3.

Additional genomic regions were identified when patients were stratified according the use of surgery for treating of their cancer. On 7q21.3-q22.3 a 12.2 Mb large gain was overrepresented in patients without surgical treatment. A short 0.33 Mb overlapping gain region on 20q11.22 was also significantly more frequent in patients with no surgical treatment.

No significant overrepresentations of gains or losses were found when tumors were stratified according to their size in TNM classification.

The HPV genetic material was present in 5 samples out of 64. A more detailed characterization of these samples is presented in Table 4.

**Table 4:** Clinical characteristics of patients with HPV positive tumor DNA samples

Gender	Age at diagnosis	Tumor location	TNM classification	Histology	Treatment	Status
male	67	oral cavity	pT4 N0	grade 3	surgery, radiotherapy	deceased
male	58	oropharynx	pT4 pN2 M1	grade 1	surgery, radiotherapy	alive
male	51	oropharynx	T4 N1	grade 2	chemotherapy, radiotherapy	alive
male	60	oral cavity	pT4 pN2	grade 1	surgery	deceased
male	55	oropharynx	pT4 pN2	grade 3	surgery, chemotherapy, radiotherapy	deceased

## Discussion

**Fig. 1:**
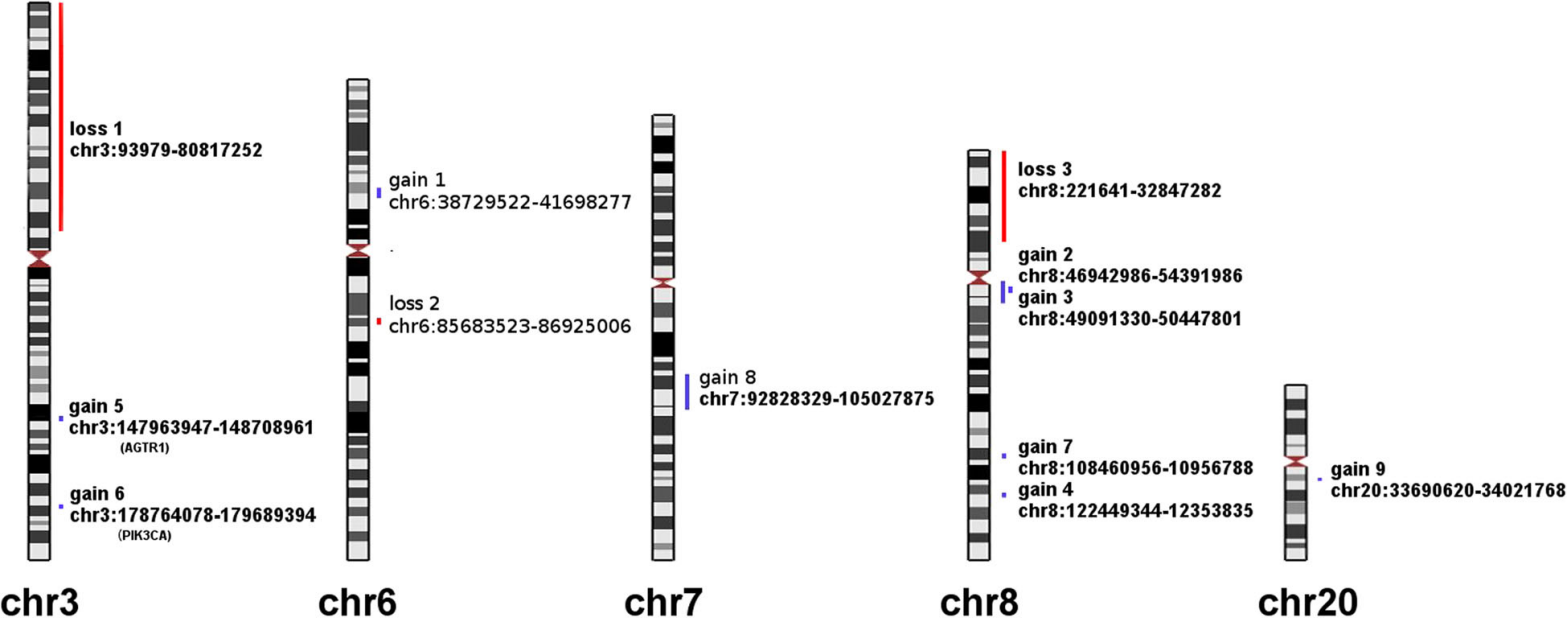
Chromosomal locations of genomic regions associated with various subgroups of patients

The role of gains and losses of large chromosomal regions in cancer has been and still is a major field of research. In our study we analyzed possible associations between CNVs and two clinically distinct groups of head and neck squamous cell carcinomas, early disease and locoregionally advanced cancer. We identified 12 regions from 5 different chromosomes to be significantly overrepresented in 4 different subgroups stratified according to selected clinical parameters (Table 3). We consider overrepresentation of gains of two regions from chromosome 3 in cases with confirmed cancer in local lymph nodes to be important. In the first instance, the region of interest contains a known oncogene, *PIK3CA* (Gain 6, Table 3, Fig. 1), which has a well-established role in cancer development [18]. The product of this gene is a catalytic unit of the PI3K kinase and in most cancer cases harbors activating mutations [19]. The signaling pathway, PI3K/AKT, is also very frequently mutated in HNSCCs [20]. Copy number gain of the *PIK3CA* region has been documented to be important in HNSCC, but the reported finding [21] is somewhat contradictory to our results because it conferred poor survival to metastatic HNSCCs with unaffected lymph nodes, whereas our study showed more *PIK3CA* copy number gains in cancers with affected lymph nodes (Table 3). Earlier studies have identified the *PIK3CA* gains in the HNSCCs [22], as well as in other major cancers; i.e. breast cancer [23], lung cancer [24] or urothelial cancer [25]. However, PIK3CA gains influencing the spreading of metastases to the lymph nodes have not been reported for HNSCCs. The *PIK3CA* alterations appear to be present in squamous cell carcinomas regardless from which organ they originate [26]. Not surprisingly we were able to associate a member of a gene signature specific for squamous cell carcinoma with a clinically distinct subtype. It is important to note that the aggressive behavior of tumors with altered PIK3CA gene function is not the only possible consequence, because the role as good prognostic marker is also known for the activated PI3K/AKT signalling pathway [27].

Secondly, the overrepresentation of a region containing the gene for angiotensin II receptor type 1 (*AGTR1*) (Gain 5, Table 3, Fig. 1) in HNSCC cases with metastases in local lymph nodes was observed. Although this association was weaker in comparison to previously described *PIK3CA* association, it nevertheless identified a very interesting gene, *AGTR1*, as a tumor biomarker for HNSCC. This observation is in line with the increased expression of *AGTR1* in head and neck cancer [28]. Also, the *AGTR1* gene was found to be implicated in other important cancers, i.e. colorectal or breast cancer [29, 30]. The association of the *AGTR1* gene with advanced head and neck cancer is important because it identifies an additional target for chemotherapy as there are several different blocking agents already in use for the treatment of hypertension. Such therapeutic approach was proposed for breast cancer cases overexpressing AGTR1 [31] and the effect of the drugs is known from in-vitro cell line experiments [32]. Because comparable data is not available for head and neck cancers, further inquiries into AGTR1 expression changes and underlying genetic alterations may help establish this gene and its signalling pathway as a legitimate therapeutic target.

We found gains of two genomic regions (Gain 8 and 9, Table 3, Fig. 1) to be overrepresented in a subgroup of patients who have not undergone radical surgery. These individuals either had an inoperable tumor, were in very poor general condition with additional other ailments, or they declined the operation because the procedure was deemed too mutilating. Their tumors were all large lesions with local invasion and extensive lymph node involvement. The first region from chromosome 7 (gain 8) overlaps with the 7q21 chromosomal region previously reported to be present in additional copies in head and neck cancer [33]. Although it contains many interesting genes which are continuously studied with respect to their role in cancer development in an ever increasing body of research, a well-established biomarker has not emerged so far. The second region (Gain 9) is from 20q which has also been implicated in HNSCC [34]. The Gain 9 is a much smaller region than Gain 8 region, but it contains several genes which may contribute to aggressive tumor growth when they are overexpressed. Specific reports for HNSCC may not be available for all the genes of interest but published studies clearly document the cancer involvement for *MMP24*, *EIF6 FAM83C* and *GDF5* [35–38]. These genes together with the large number of genes from the Gain 8 region warrant further analysis because they are potential biomarkers for aggressive tumor growth.

The remaining genomic regions were all overrepresented in subgroups of patients with a less aggressive or less advanced form of HNSCC. The gains (Gain 1, 2, 3, 4, 7) and losses (Loss 1, 2, 3) are presented in Table 3 and showed in Fig. 1. Such significant increase, that is not associated with a higher malignancy but with less malignant cases of HNSCC may indicate a possible role in earlier phases of cancer progression. Among the many genes included in the described variations, several genes were already studied for their role in cancer development. For most of these candidate genes the available information does not derine from studies on HNSCC but from studies focusing on other types of cancer. In less advanced cancers with stage I and II the overrepresented region gain 1 contained at least 2 genes of interest: overexpressed *TREM2* gene known to be implicated in cell proliferation, was observed in early cases of esophageal cancer [39, 40] whereas the *TFEB* gene, a transcription factor for lysosomal biogenesis, is part of a well- documented cancer specific translocation t(6;11) [41, 42]. The genomic region Gain 2 from chromosome 8 also contains additional genes important for cancer development. Thus, the overexpression of *MCM4* gene was detected in laryngeal squamous cell carcinoma and this gene was also implicated in other cancers because it codes for an important component of the DNA replication machinery [43, 44]. Inside the genomic region Gain 2 a continuous segment, named Gain 3, was obverved, which showed an even a higher level of association and contained the *SNAI2* gene. This is a transcription factor with a demonstrated role as an oncogene in HNSCC [45]. Notwithstanding, we did observe overrepresented gains in less malignant cases (stage I & II) while the gene was found to contribute to poor disease survival [46]. This is contradictory but it may be that an overexpressed *SNAI2* is required early in HNSCC development to gain malignant potential.

The largest genomic region exhibiting significant association comprises almost the entire short arm of the chromosome 3 (Loss 1, Table 3, Fig. 1). The loss of this region was overrepresented in well-differentiated grade 1 classified tumors. Thus, our observation identifies a well documented chromosomal aberration as a biomarker for a subgroup of tumors which retained good differentiation. As noted previously, earlier studies identified frequent presence of the 3p deletion in head and neck cancers [47, 48]. Then the 3p deletion was described in early displastic lesions [49]. More recently, when *p53* mutations were detected in combination with 3p deletions worse survival was observed in such cancer cases [50]. Therefore our result identifies a novel facet of this particular genetic alteration, namely its presence in well differentiated HNSCCs, which has not been reported so far.

Furthermore, additional three genomic regions were also overrepresented in well differentiated grade 1 tumors (Table 3, Fig. 1), whereas the Gain 4 region comprises only of 2 possible genes/transcribed regions (*HAS2-AS1, LOC105375734*) - with little available information. The loss of a region from chromosome 6 (Loss 2) overlaps with a 6q14 region previously described to be deleted in HNSCC [51]. This region was studied for its role in many different cancer types but it was only recently demonstrated that the 2 genes coding for small nuclear RNA, *SNORD50A* and *SNORD50B*, directly affect the activity of K-Ras, because both snoRNAs bind to it and when deleted, K-Ras activity is increased [52]. Finally, we observed a deletion of the large part of the short arm of chromosome 8 (Loss 3) to be significantly more frequent in grade 1 tumors than in poorly differentiated tumors. This particular aberration is also well known in HNSCC; however, we detected an overabundance in a well differentiated subgroup of tumors which appears to be a novel observation [47, 48]. The 8p deletions are characteristic for HNSCC but their role is not well understood. Although from other cancers a more malignant behavior was observed when its presence was detected [53].

Our study found several genomic regions (Gain 1, 2, 3, 4, 7, Loss 1, 2, 3; listed in Table 3, presented in Fig. 1) with CNVs to be more frequent in tumor subgroups with less malignant properties. Such findings suggest that certain genetic variations may have a more transitory role in cancer progression. They may be crucial for the tumor to reach a certain stage in its development and are then less important for the further disease course. It is also possible that such variations remain present in the tumor but their existence is masked by the clonal expansion of other cancer cells. Consequently when tumor samples are obtained for a study, relevant sections of tumor may not be included and a single sample from large, advanced tumor may not contain all cancer cell clones. Also the aCGH analysis requires the use of extracted DNA from a highly heterogeneous tumor sample where low percentage CNVs may be masked by normal DNA. The protocol implemented in our study certainly introduced further variability into the analysis of HNSCCs, which are considered highly heterogeneous tumors. As a consequence, it is not particularly surprising that associations were not observed for the most common and typical genomic regions implicated in HNSCC. I.e., the gain of 11q13 region containing the *CCND1* gene, was the most frequent CNV in our sample for which we did not detect any significant association (data not shown). Several other genomic regions with important genes showed frequent CNVs (i.e. 7p12, *EGFR* gene) but associations were not present (data not shown) [54]. Such findings can be attributed to a small sample size available for this study which in combination with highly heterogeneous clinical presentation of HNSCC restricts the ability to detect significant associations between genetic markers and clinical parameters. Consequently, we were not able to observe significant differences in survival and this analysis was omitted from the study. In addition, we detected HPV DNA in 5 out of 64 samples (clinical data presented in Table 4) which is significantly below the rate of approximately 20% reported HNSCC cases in Slovenia [55]. Therefore, it was not possible to detect CNVs that are specifically associated with the HPV infection and HNSCC.

## Conclusions

This analysis compared frequencies of genomic copy number variations in subgroups of HNSCC patients which were stratified according to clinical parameters characteristic for early or advanced cancer. We identified gains of genomic regions from 3q containing *PIK3CA* and *AGTR1* genes with significantly higher frequencies in cases with lymph node involvement. The cancer cases which were not treated with surgery also harbored gains of 7q21 and 20q genomic regions significantly more often. Interestingly, other gaines and losses with significant associations were overrepresented in subgroups defined by parameters characteristic for early HNSCC. Nevertheless, many different genes could be implicated in HNSCC development and they all represent a potentially important source of biomarkers useful for clinical management of this cancer and targets for further research.

## Abbreviations

aCGH: Array comparative genomic hybridization
CNV: Copy number variation
HNSCC: Head and neck squamous cell carcinoma
HPV: Human papilloma virus
